# Aberrant modulation of the BRCA1 and G1/S cell cycle pathways in alcoholic hepatitis patients with Mallory Denk Bodies revealed by RNA sequencing

**DOI:** 10.18632/oncotarget.6382

**Published:** 2015-11-25

**Authors:** Hui Liu, Ming Gong, Barbara A. French, Guanghong Liao, Jun Li, Brittany Tillman, Samuel W. French

**Affiliations:** ^1^ Department of Pathology, LABioMed at Harbor UCLA Medical Center, Torrance, CA, USA; ^2^ Department of Pediatrics, LABioMed at Harbor UCLA Medical Center, Torrance, CA, USA

**Keywords:** liver disease, cell cycle arrest, balloon hepatocyte, p15, p21, Pathology Section

## Abstract

Mallory-Denk Bodies (MDBs) are prevalent in various liver diseases including alcoholic hepatitis (AH) and are formed in mice livers by feeding DDC. Liver injury from alcohol administration causes balloon hepatocytes and MDB formation impeding liver regeneration. By comparing AH livers where MDBs had formed with normal liver transcriptomes obtained by RNA sequencing (RNA-Seq), there was significant upregulation of BRCA1-mediated signaling and G1/S cell cycle checkpoint pathways. The transcriptional architecture of differentially expressed genes from AH livers reflected step-wise transcriptional changes progressing to AH. Key molecules such as BRCA1, p15 and p21 were significantly upregulated both in AH livers and in the livers of the DDC re-fed mice model where MDBs had formed. The increase of G1/S cell cycle checkpoint inhibitors p15 and p21 results in cell cycle arrest and inhibition of liver regeneration, implying that p15 and p21 could be exploited for the identification of specific targets for the treatment of liver disease. Provided here for the first time is the RNA-Seq data that represents the fully annotated catalogue of the expression of mRNAs. The most prominent alterations observed were the changes in BRCA1-mediated signaling and G1/S cell cycle checkpoint pathways. These new findings expand previous and related knowledge in the search for gene changes that might be critical in the understanding of the underlying progression to the development of AH.

## INTRODUCTION

The liver acts as a hub for metabolic reactions to keep a homeostatic balance during development and growth. Reactive oxygen and nitrogen species (ROS and RNS, respectively) are products of normal cellular metabolism. Oxidative stress has been proposed to be crucially involved in alcoholic liver disease (ALD) including alcoholic hepatitis (AH) [1]. The pathogenesis of ALD has been linked to cell cycle arrest, which inhibits liver cell regeneration. Liver cells enter the cell cycle, G1 phase, and progress to S phase in response to stress. The DNA damage response pathways activate the checkpoint to arrest the cell cycle transiently, promoting DNA repair and inducing cell senescence or apoptosis [2]. The role of the cell cycle Cip/Kip family members p21 (Cdkn1a) and p27 (Cdkn1b) are over-expressed in AH and in rats chronically fed ethanol [3, 4]. P21 and p27 over-expression inhibits the regeneration of the liver in rats after partially hepatectomy [3]. Cdkn2a and Cdkn2b genes encode p16 and p15 inhibitors of the G1/S phase of the cell cycle [5], and derepression of Cdkn2a has been suggested to induce cell death through downstream targets of p53, including p21 and Wig1 [6].

Mallory-Denk bodies (MDBs) are found in 70% to 75% of patients with AH [7]. MDBs are composed of intracellular aggregations of misfolded proteins in ballooned hepatocytes. They consist of abnormally phosphorylated, ubiquitinated, and cross-linked keratins 8 and 18 (K8/K18) and non-keratin components [8]. A major player that determines MDB formation is the ballooned hepatocyte. MDB-forming hepatocytes stain positive for numerous markers of preneoplasmic change [9]. MDB-forming hepatocytes represent volume increase (hydration) of the hepatocytes. MDBs form due to the failure of the 26S proteasome protein quality control system which leads to aggresomes composed of cytokeratins (CKs) and the undigested proteins such as heat shock proteins (HSPs), Ub, proteasome subunits, tubulin, and the ubiquitin-binding protein p62 [10]. The swelling of the balloon cell cytoplasm is due to osmotic effect of the accumulation of these undigested proteins.

MDBs develop in the liver of DDC re-fed mice. In the DDC fed mouse model where liver cells proliferate, MDBs form and later, after DDC withdrawal (DDC primed hepatocytes), hepatocellular carcinoma (HCC) develops [11, 12]. It was found that the pathogenesis of MDBs is associated with the downregulation of ufm1-conjugation system (Ufmylation) and FAT10-conjugation system (FATylation) pathways involved in protein quality control [13], and upregulation of the NFκB-CXCR4/7 pathway [14] in MDB-forming AH patients.

Several research strategies have been used to study the pathogenesis of AH. However, few have provided specific mechanisms associated with MDB development in AH. High-throughput RNA-sequencing (RNA-Seq) is a recently developed technology that provides new strategies for analyzing the functional complexity of a transcriptome [15, 16]. To explore the mechanisms (e.g., signaling pathways) that mediate the initiation and progression of AH and cell cycle arrest, the RNA-Seq (Illumina sequencing) was performed to evaluate the patients' overall liver transcriptome. Aberrant modulations of the BRCA1-mediated signaling and G1/S cell cycle checkpoint in AH livers where MDBs were formed was found. Also the key candidate biomarkers (BRCA1, p15 and p21, etc.) were markedly overexpressed in human and in the livers of DDC re-fed mice forming MDBs. Combining this new data with the previous publications, the events that may drive liver MDB formation and cell cycle arrest in AH livers and subsequent tumor progression were re-evaluated.

## RESULTS

### Expression shift profile between alcoholic hepatitis (AH) livers with Mallory-Denk Bodies (MDBs)

**Figure 1:**
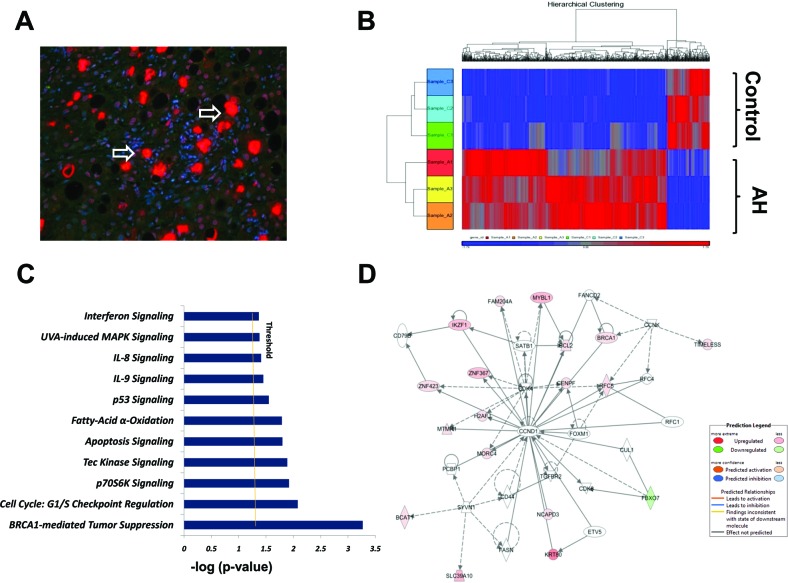
High-resolution transcriptsome analysis of human AH livers with MDBs present **A**. A representative immunohistochemical staining section (4 μm) from a human AH biopsy, double stained with Ub (red) and DAPI (blue). The white arrow indicates the balloon cells that had formed MDBs. ×20. **B**. 2-Dimensional hierarchical clustering using the top 798 selected genes discriminating 3 alcoholic hepatitis livers from 3 normal control livers. Each column represents a subject and each row a gene probe set. Probe set signal values were normalized to the mean across the livers. The relative level of gene expression is depicted by heat map from the lowest (blue) to the highest (red), according to the scale shown on the top. Respective gene symbols are indicated on the top side. **C**. Significance levels of functions of differentially expressed hepatic genes in AH livers with MDBs. Threshold was set at p=0.05 and indicated as -log (*p* value) on the X-axis. Y-axis shows functions of differentially expressed genes. **D**. The network was algorithmically constructed by Ingenuity Pathway Analysis (IPA) software on the basis of the functional and biological connectivity of genes. The network is graphically represented as nodes (genes) and edges (the biological relationship between genes). Red and green shaded nodes represent up- and down regulated genes, respectively; others (empty nodes) are those that IPA automatically includes because they are biologically linked to these genes based on the evidence in the literature. Top ranked network generated by IPA with cell cycle modulated genes (score 16, n=35 associated genes, *P* < 0.05). This network is centered around the canonical cell cycle-related molecules cyclin D1 (CCND1). Meanings of node shapes and edges are indicated in the legend within the figure.

To uncover molecular mechanisms involved in Mallory-Denk Bodies (MDBs) pathogenesis in alcoholic hepatitis (AH), the genomic profile was compared using RNA sequencing (RNA-Seq) in a group of archived AH liver biopsies who had formed MDBs and archived normal liver controls with normal hepatic function, after having their informed consent. The immunohistochemical staining for human AH livers with MDBs was successfully used in prior biopsy sections tested [4, 13, 14]. A representative AH biopsy in which the balloon cells had formed MDBs is shown (Figure 1A). The Principal-Component Analysis (PCA) Mapping indicates that the three samples in the AH group (red color) clustered together and the three samples in the normal liver group (blue color) clustered together (Supplementary Figure 1), suggesting a clear difference between these two groups. The RNA-Seq analysis revealed 798 differentially expressed genes (DEGs) differentiating the AH livers from normal livers (false discovery rate (FDR)-adjusted *P*-value< 0.05 and fold-change ≥2). The two-dimensional hierarchical clustering, using the 798 DEGs, clearly showed the degree of separation between the AH livers with MDBs and normal control livers (Figure 1B). Of these DEGs, 660 were specifically upregulated (Supplementary Table S2), and 138 genes were specifically downregulated (Supplementary Table S3). The fold-changes in the AH livers ranged from −16.12 to 1069.6 (Supplementary Table S2, S3).

MDBs contain cytokeratin (CK) and heat shock proteins (HSPs) [17, 18]. Several molecules related to MDB formation included HSPA2 (heat shock 70kDa protein 2), KRT80 (Keratin80), and HSPA12A (heat shock 70kDa protein 12A) were also discovered in the RNA-Seq database and were significantly upregulated (Supplementary Table S2). The protein degradation pathway and TLR signaling are crucial for liver MDB formation in AH and non-alcoholic steatohepatitis (NASH) [13, 14]. The previously identified set of genes reported was compared with the expression pattern in the RNA-seq database. As expected, mRNA expression determined by RNA-Seq for key molecules involved in Ufmylation, FATylation and TLR signaling, such as UBD (FAT10; 9.041107 fold; *p*=0.007061), ubiquitin fold modifier 1 (Ufm1) (−2.121568 fold, *p*=0.002144), UfSP1 (−3.161889 fold, *p*=0.007848) and TLR4 (8.27599 fold, *p*=0.003397) (Supplementary Tables S2, S3) were simultaneously expressed when compared to the mRNA expression determined by quantitative real-time PCR (qRT-PCR). This supported the previous conclusions.

### Functional analysis of the DEGs identified by RNA-Seq

For all the DEGs among AH and normal control liver groups, pathway analyses were constructed using ingenuity pathway analysis (IPA). IPA demonstrated that a total number of 19 pathways were defined with at least three genes related to each pathway (Supplementary Table S4). To define the most significantly changed pathways, the number of differentially expressed genes involved in each pathway were prepared as a standard. Eleven pathways with prominent changes in this analysis are shown in Figure 1C. Among these differentially regulated pathways, the BRCA1-mediated tumor suppression and G1/S cell cycle checkpoint (FDR-adjusted *P*-value< 0.05, respectively) were the two most significantly changed canonical pathways thus identified (Table 1). The p70S6K signaling, Tec kinase pathway and apoptosis signaling are the other three significantly altered canonical pathways (Supplementary Table S4). In addition, the top ranked co-expression network for “Gene Disease and Function” was mainly involved in Cell Cycle disorders (Figure 1D), including the genes encoding for regulators, for example, BRCA1 and KRT80 (Supplementary Table S5). The “Cell death and Survival” is another enriched co-expression network (Supplementary Table S5). Further functional analysis for “Gene Disease and Function” classification by IPA revealed that all up- and down regulated DEGs were mainly associated with increased cell viability (z-score=2.47; Supplementary Table S6), which is in accordance with the enriched biological process of the canonical pathways.

**Table 1:** Most representative canonical pathways deregulated in AH livers with MDBs

IPA category	Pathway -log (P-value^a^)	Gene symbol	Molecules in dataset	Ratio
BRCA1-mediated	3.27E00	*BRCA1*	Breast cancer 1, early onset	1.41E-01
tumor suppression		*BRCA2*	Breast cancer 2, early onset	
		*CDKN1A*	Cyclin-dependent kinase inhibitor 1A (p21, Cip1)	
		*BRCC3*	BRCA1/BRCA2-containing complex, subunit 3	
		*E2F3*	E2F transcription factor 3	
		*RFC5*	Eplication factor C (activator 1) 5, 36.5kDa	
		*ATM*	ATMserine/threonine kinase	
		*FANCG*	Fanconi anemia, complementation group G	
		*SLC19A1*	Solute carrier family 19 (folate transporter), member 1	
		*FANCA*	Fanconi anemia, complementation group A	
G1/S cell cycle	2.08E00	*CDKN2B*	Cyclin-dependent kinase inhibitor 2B (p15)	1.11E-01
Checkpoint		*CDKN1A*	Cyclin-dependent kinase inhibitor 1A (p21, Cip1)	
		*CDK2*	Cyclin-dependent kinase 2	
		*ATM*	ATMserine/threonine kinase	
		*E2F3*	E2F transcription factor 3	
		*NRG1*	Neuregulin 1	
		*HDAC11*	Histone deacetylase 11	
		*SUV39H1*	Suppressor of variegation 3-9	

Abbreviations: FDR, false discovery rate; IPA, ingenuity pathway analysis.

a Fischer's exact test was used to calculate the P-value, determining the probability that the association between the genes in the data set and the canonical pathway is explained by chance alone. To account for multiple canonical pathways tested by IPA, the FDR option was used (FDR<0.1).

### The expression of differential genes in the livers of patients and mice fed DDC

**Figure 2:**
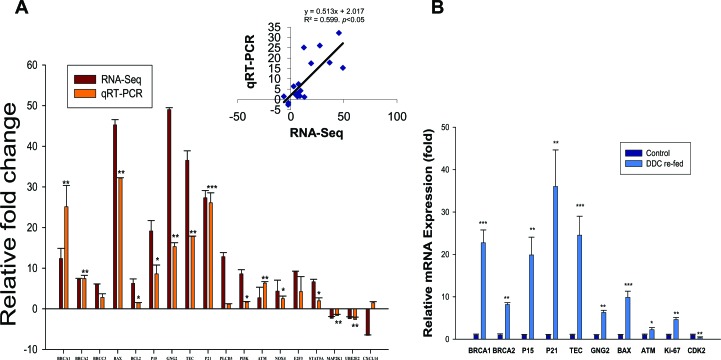
The expression analysis of differential genes in the livers of AH and mice fed DDC **A**. Comparison of transcript expression in terms of fold change was measured by RNA-Seq and qRT-PCR. The transcript expression fold changes measured by RNA-Seq and qRT-PCR are indicated by violet and orange red columns, respectively. Asterisks on the qRT-PCR values indicate significant differences between normal livers and AH livers with MDBs. n=3. **B**. Induction of BRCA1, BRCA2, P15, P21, TEC, GNG2, BAX, ATM, Ki67 and CDK2 in the livers of DDC re-fed mice. *p<0.05, **p<0.01, and ***p<0.001.

A total of eighteen genes with various fold changes from the above mentioned top 5 pathways (Supplementary Table S4) were selected and quantified to verify the RNA-Seq data by qRT-PCR on all AH livers and normal liver biopsies. As shown in Figure 2A, 15 genes were significantly upregulated, and 2 genes had slightly downregulation expression, which is consistent with the RNA-Seq results. There was discordance between the RNA-Seq and the qRT-PCR in the relative expression levels for only one of eighteen genes (CXCL14) (Figure 2A). Furthermore, Pearson's correlation coefficient was performed to validate our results. A statistically significant correlation (R=0.599, *p*< 0.05) between the qRT-PCR and RNA-Seq results was observed for all tested genes (Figure 2A), indicating a relatively high concordance between these two databases.

DDC feeding was used to induce MDB formation in mice. Mice liver tissue isolation was performed from mice fed the control diet and mice re-fed DDC for 7 days as described earlier [19]. The RNA-Seq analysis of markedly changed genes was further examined in the livers of DDC re-fed mice using qRT-PCR analysis. The mRNA expression of BRCA1, BRCA2, p15, p21, Tec, guanine nucleotide binding protein (G protein), gamma2 (GNG2), Bax, ATM and Ki67, which are substantially upregulated in the AH livers, were all upregulated to various degrees (Figure 2B). The mRNAs of BRCA1, p21, p15 and Tec were significantly upregulated when compared to other mRNAs. Among these, p21 showed a very high 40-fold induction in the livers of DDC re-fed mice. Other molecules including BRCA1, p15 and Tec mRNAs were induced to approximately 22-, 20- and 25-fold (respectively, *p*<0.01) in this test. CDK2 mRNA, however, is significantly downregulated in the livers of DDC re-fed mice (Figure 2B), similar to the observation in the AH livers with MDBs determined by RNA-Seq (Table S3). These data clearly suggested that these key regulators might be involved in liver MDB formation.

### Hyperactivation of the BRCA1-mediated pathway in the AH patients with MDBs

The IPA revealed that the most changed pathway (z-score=1.89) is the BRCA1-mediated signaling pathway in AH where MDBs had formed (Table 1 and Supplementary Table S4). A schematic diagram of the activated BRCA1-mediated signaling pathway derived using IPA was shown (Supplementary Figure 2). The extremely high expression and induction in the AH livers and DDC re-fed mice led to the possibility that BRCA1 is a central element in this pathway and plays an important role in the initiation and progression of liver MDB formation.

**Figure 3:**
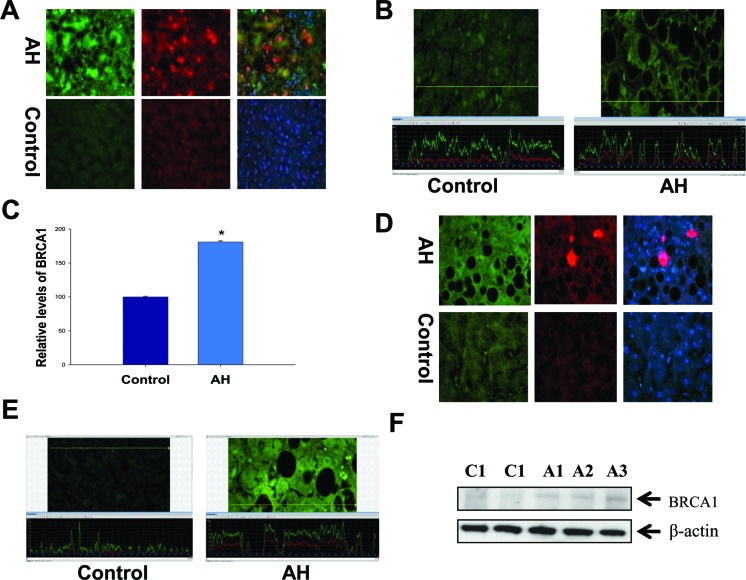
Altered modulation of the BRCA1-mediated pathway in the liver of AH livers with MDBs **A**. The liver sections from AH livers and controls were double stained with antibodies to BRCA1 (green), Ub (red) and DAP1 (blue). The MDBs stained positive for both Ub and BRCA1. ×520. **B**. The liver sections from patients with AH (right) and controls (left) were stained with an antibody against BRCA1. The hepatic cells without MDBs from patients with AH demonstrated greater intensity for BRCA1 compared to controls. A representative screen snip was obtained from morphometric screen. The fluorescence intensity was traced along the yellow line in the top panel and shown as a green tracer in the bottom picture. **C**. Quantification of fluorescence intensity of the immunoactivity of BRCA1 in B (n=3 patients; 25 cells/liver sections from each patients). **D**. The liver sections from AH livers and controls were double stained with antibodies to BRCA2 (green), Ub (red) and DAP1 (blue). The MDBs stained positive for both Ub and BRCA2. ×520. **E**. The liver sections from AH (right) and controls (left) were stained with an antibody against BRCA2. A representative screen snip obtained from the morphometric screen showed the hepatic cells without MDBs from AH livers showed greater intensity for BRCA2 compared to controls. **F**. Western blot analysis of BRCA1 in AH (A1, A2 and A3) and control biopsies (C1 and C2). Data represent mean values ±S.E.M. *p<0.01.

The immunohistochemical staining was performed in all AH livers tested in this RNA-Seq, which showed that MDB containing balloon liver cells in AH liver biopsies had increased staining intensity of the cytoplasm for BRCA1 (Figure 3A). The morphometric screen hunters were then used to measure and visualize the intensity of nuclear staining of BRCA1 in the nuclei of hepatic cells without MDBs compared to the nuclei of neighboring normal liver cells. The results showed that the nucleus in hepatic cells without MDBs stained with more intensity compared to the nucleus in the normal liver cells (Figure 3B). The yellow line on the photomicrograph runs over the nuclei indicating where the intensity was measured. The immunoreactivity intensity of BRCA1 in all tested livers with AH was 180% of that in controls (*p*<0.01) (Figure 3C), where twenty-five cells from liver sections of each patient were analyzed. Also, BRCA2 had increased staining intensity of the cytoplasm in MDB containing balloon liver cells of AH biopsies determined by immunohistochemical staining (Figure 3D). Similar to BRCA1, the morphometric screen hunters showed that the nucleus in hepatic cells without MDBs stained with more intensity for BRCA2 compared to the nucleus in the normal liver cells (Figure 3E). The protein level of BRCA1 in MDB-forming hepatic cells was evaluated by Western blot. A marked increase in the AH livers compared with normal controls were observed (Figure 3F).

Other components of the BRCA1-mediated pathway with notable upregulation included ATM, p21, E2F (more than a 2-fold change, Supplementary Table S2) beside RFC, where mRNA was downregulated (Supplementary Table S3). All these results imply that the pathogenesis of liver MDB formation is linked to upregulation of the BRCA1-mediated signaling.

### The altered modulation of G1/S cell cycle in AH patients with MDBs present

**Figure 4:**
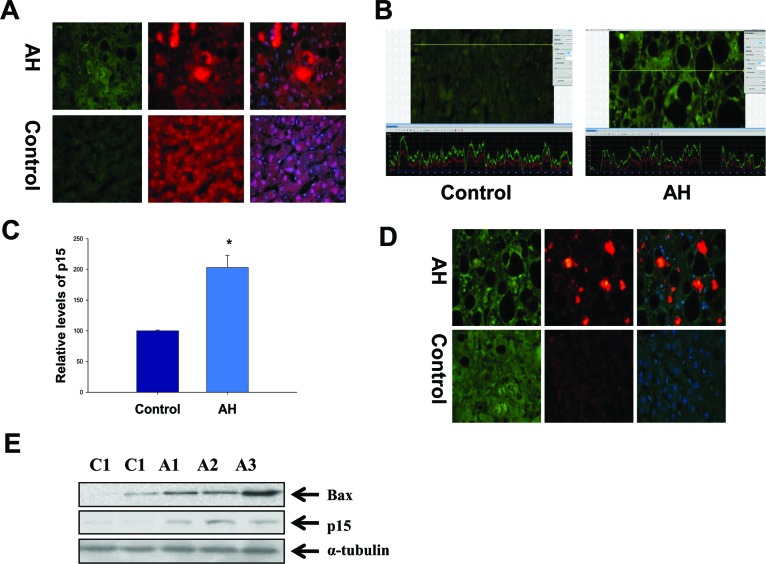
Hyperactivation of the G1/S cell cycle checkpoint in the liver of AH livers with MDBs **A**. Liver sections from AH patients and controls were double stained with antibodies to p15 (green), Ub (red) and DAP1 (blue). The MDBs stained positive for both Ub and p15. ×520. **B**. The liver sections from AH (right) and controls (left) were stained with an antibody against p15. A representative screen snip obtained from the morphometric screen demonstrated the hepatic cells without MDBs from AH livers have greater intensity for p15 compared to controls. **C**. Quantification of fluorescence intensity of the immunoactivity of p15 in B (n=3 patients; 25 cells/liver sections from each patients). **D**. The liver sections from AH and controls were double stained with antibodies to Bax (green), Ub (red) and DAP1 (blue). The MDBs stained positive for both Ub and Bax. ×520. **E**. Western blot analysis of p15 and Bax in AH (A1, A2 and A3) and normal biopsies (C1 and C2). Data represent mean values ±S.E.M. *p<0.001.

Another markedly changed pathway in the RNA-Seq database determined by IPA was the G1/S cell cycle checkpoint in AH livers with MDBs (Table 1). P21, a cell cycle inhibitor at G1/S growth phase, was significantly induced in the livers of AH and DDC re-fed mice (Figure 2A and 2B), and was shown to be expressed in numerous hepatic nuclei in the liver biopsies from patients with AH as reported previously [4]. It was further observed that p15, a member of the Ink4 family mediating G1 cell cycle arrest [20] was prominently induced in the livers of AH and DDC re-fed mice, causing speculation as to its role in cell cycle arrest. Similar to p21, immunohistochemical staining showed that balloon hepatocytes with MDBs in AH liver biopsies were expressing p15 in the cytoplasm (Figure 4A). The intensity of nuclear staining for p15 in the nuclei of hepatic cells without MDBs was compared to the nuclei of neighboring normal liver cells, which showed elevated intensity in contrast to the nucleus in the normal control liver cells (Figure 4B). The immunoreactivity intensity of p15 in all AH patients tested was 203% (25 hepatic cells without MDBs/liver section from each patient) of that in control subjects (*p*<0.01) (Figure 4C). The p15 protein was significantly increased by Western blot analysis in AH biopsies (Figure 4E). This is analogous to the mRNA level determined by qRT-PCR and RAN-Seq (Figure 2A). The mRNA level of p21 expression was high in the AH livers and DDC re-fed mice.

When the cell cycle is arrested, apoptosis will occur and liver regeneration is impeded. Therefore, the immunohistochemical staining and Western blot were performed to evaluate the change of apoptosis-related molecule Bax. The results showed that Bax staining intensity was increased in the liver cytoplasm (Figure 4D), and also was significantly increased in the AH biopsies compared to the normal control livers (Figure 4E). These data indicate a possible role of the G1/S cell cycle checkpoint in the reduced liver regeneration observed in AH pathogenesis.

## DISCUSSION

**Figure 5:**
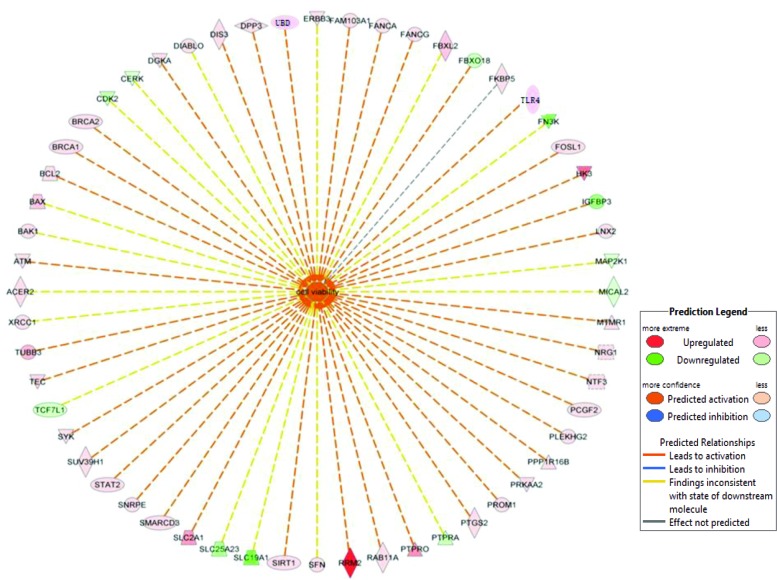
List of genes involved in increased cell viability in AH livers with MDBs The genes in the red box illustrate differentially expressed genes between the AH livers and controls in the RNA-Seq analysis. The network was algorithmically constructed by IPA software on the basis of the disease and functional connectivity of genes. Red and green shaded nodes represent up- and down regulated genes, respectively; Top ranked disease and function network generated by IPA with cell viability related genes (n=56 associated genes, z-score=2.47). Meanings of node shapes and edges are indicated in the legend in the figure.

**Figure 6:**
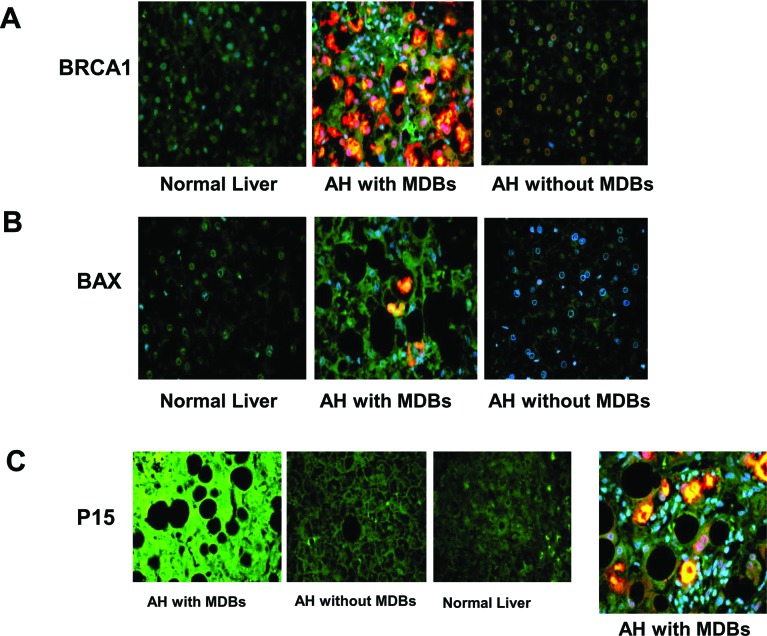
**The liver sections from AH patients with MDBs, AH patients without MDBs and controls were double stained with antibodies to BRCA1 A.,** Bax **B**. and p15 **C**. together with Ub (red) and DAPI (tricolor). The AH liver sections with MDBs stained with greater intensity for BRCA1, Bax and p15 compared to the controls and AH livers without MDBs. ×520.

In this study, we found that the discriminated expression genes mainly involved in the aberrant modulation of the BRCA1-mediated signaling and G1/S cell cycle checkpoint. The specificity of these pathways in AH livers compared with normal livers were confirmed with GSEA as previously reported [21]. Further disease and functional analysis for DEGs revealed obviously increased cell viability of tumor cell generation (Figure 5), reflecting step-wise transcriptional changes from AH progressing to HCC in response to liver injury from alcohol administration. Recent microarray analysis identifies TNF superfamily receptors are overexpressed in AH [22]. Some genes of TNF family such as TRAF1 (8.12 fold; *p*=0.006), TNFRSF1B (2.77 fold; *p*=0.023) and FASTKD5 (5.86 fold; *p*=0.014) are also overexpressed in our RNA-Seq analysis (Supplementary Table S2). The different observations between previous microarray and our RNA-Seq results may be from the difference in the samples used. Another reason is that the basic justification for our approach is to focus on individual MDB-forming balloon liver cells rather than including all the different cell types in the liver. The next goal is to discover novel molecular biomarkers involved in AH with MDBs. What was found was (a) higher BRCA1 mRNA and protein levels, (b) increased p15 and Bax mRNAs and protein levels, and (c) enhanced p21 and Tec mRNA levels. Interestingly, the immunostaining showed that the AH liver biopsies with MDBs had increased staining intensity than the livers without MDBs and normal livers for BRCA1, BAX and p15 (Figure 6). All these candidate biomarkers were further confirmed in MDB-forming AH livers.

Comprehensive pathway analysis revealed that the BRCA1-mediated pathway is the most enriched altered pathway (Supplementary Table S4) in AH livers with MDBs. BRCA1 is established as a tumor suppressor and functions primarily by maintaining genome integrity [23]. Interestingly, it showed that multiple phosphorylations may activate BRCA1 function in response ROS and BRCA1 maybe an ROS sensor [23]. The BRCA1/BARD1 complex can stabilize p53 and induce cell cycle protein p21 in response to interferon gamma (IFNγ) [24]. Tumor necrosis factors-α (TNFα) and IFNγ strongly induce the FAT10 and LMP2 expression, resulting in a decrease in protein degradation and liver MDB formation [13, 25].

Hepatocyte regeneration is an important part of the repair process during the recovery stage of alcoholic liver disease. Progression through the G1 phase is facilitated by the D-type cyclins (D1, D2, D3), which form active holoenzymes with cyclin-dependent kinases 4 or 6 (CDK4, CDK6), and cyclin E, which activates CDK2 [26]. The S phase marker CDK2 is significantly downregulated in AH livers with MDBs and in the livers of DDC re-fed mice (Figure 2B). Subsequent phosphorylation by the cyclin E-dependent kinase results in the release of members of the E2F family of transcriptional activators from Rb [27]. The Cip/Kip family, p21, p27 and p57 can bind to complexes containing all known CDKs. P21 is found to be linked to fibrosis and an adverse liver-related outcome in alcohol-related liver disease [28]. The Ink4 family, p15, p16, p18 and p19, bind only CDK4 and CDK6, thereby mediating G1 cell cycle arrest [20]. Analysis of canonical pathways revealed that G1/S cell cycle checkpoint is another enriched upregulated pathway in AH livers with MDBs (Supplementary Tables S3). The high mRNA levels of p15 and p21, and high protein level of p15 were observed in the present study in AH livers, suggesting that cell cycle arrest occurred in AH livers due to p15 and p21 expressed in MDB-forming hepatocytes in the AH livers. Similarly, the mRNA levels of p15 and p21 were significantly upregulated in the livers of the DDC re-fed mice model, further suggesting an association of p15 and p21 with MDB formation.

**Figure 7:**
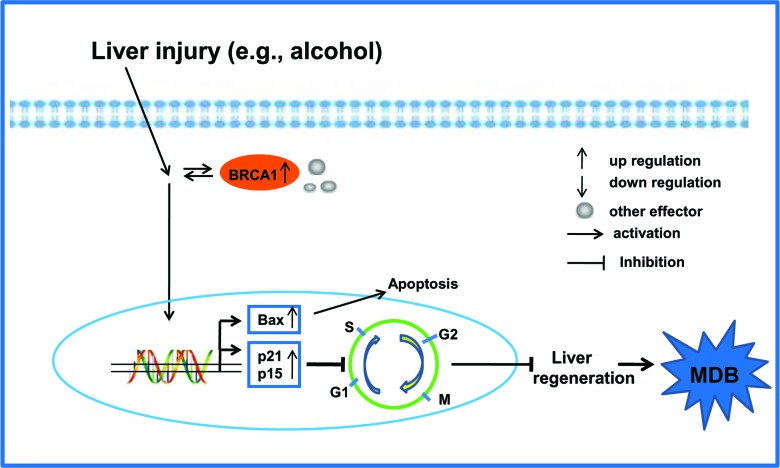
Proposed model of MDB formation mediated by the BRCA1-mediated signaling and G1/S cell cycle checkpoint in AH livers are shown Liver injury can activate the key regulator BRCA1 and other effectors in the BRCA1-mediated signaling. On the other hand, the expression of cell cycle inhibitors p21 and p15 result in the G1/S cell cycle arrest, which impedes liver regeneration and the formation of MDBs.

Based on the current information, a very large amount of BRCA1 together with other effectors (e.g., BRCA2) appeared in balloon hepatic cells from liver injury by an unknown mechanism that likely arose through adaptive evolution, which mediated downstream gene expression and prevented BRCA1-related cancer. The cell cycle inhibitors p21 and p15 are over-expressed due to the BRCA1 initiated pathway, resulting in cell cycle arrest and the impeding of liver regeneration (Figure 7). However, the key regulatory proteins of the cell cycle are governed by one or more E3 ligases (e.g., SKp2 and CRL4^Cdt2^) which fail to degrade these inhibitors (e.g., p21) of cell cycle progression during G1/S phase regulation [26, 29]. This suggests that both the mechanism of MDB formation and cell cycle arrest are a consequence of the loss of protein quality control by the 26S proteasome seen in AH since chronic ethanol feeding leads to inhibition of the 26S proteasome activity in the liver [30, 31].

The RNA-Seq data presented here also shows the remarkable alteration of p70S6K signaling (Supplementary Figure 4) and Tec kinase pathways (Supplementary Figure 5) in the AH livers with MDBs. P70S6K plays an important role in cell growth, such as proliferation and differentiation by regulating cell cycle progression and survival of cancer cells [32]. The immunostaining results for Tec showed that Tec was increased in MDB-forming hapatocytes (Supplementary Figure 6). Tec kinase signaling has been shown to control assembly and activity of the noncanonical caspase-8 inflammasome [33]. MDB formation could be an indicator of the extent of inflammasome activation [34]. These observations are suggestive of the potential effects of p70S6K and Tec kinase signaling in all the contexts where MDB-forming cells in AH face microenvironmental challenges.

In summary, the altered modulation of the BRCA1-mediated signaling and G1/S cell cycle checkpoint in AH with MDBs present may provide new explanations for the mechanisms underlying the cause for cell cycle arrest and the inhibition of liver regeneration seen in AH. The suppression of liver regeneration seen in AH is a major reason for liver failure in AH. Finally, these mechanisms could be exploited for the identification of specific targets for the treatment of liver disease.

## MATERIALS AND METHODS

### Liver biopsy specimens

Human formalin-fixed paraffin-embedded (FFPE) liver biopsies from patients who had alcoholic hepatitis (AH; n=3) were obtained from Harbor UCLA hospital archives. In all the cases liver forming MDBs were presented. Normal control livers were used for comparison. The liver biopsies used were also used in previous studies [4, 13, 35]. Also the AH livers without MDBs were used for comparison (n=3). The liver biopsy sections were 4 μm thick. The study was carried out according to the principles of the Declaration of Helsinki and was approved by our institutional ethics review board. The data were analyzed anonymously and reported.

### Mouse liver

The livers of mice in which MDB formation was induced by feeding Diethyl 1, 4-dehydro-2, 4, 6-trimethyl-3, 5-pyridine-dicarboxylate (DDC) were used to compare with the AH livers where MDBs had formed. The livers used had been used in prior studies [13, 19, 35]. All mice were treated in a humane manner as approved by the Animal Care Committee at Harbor UCLA Laboratory BioMedical Research Institute according to the Guidelines of the National Academy of Science.

### Tissue sectioning

Mice liver frozen sections were cut 5 μm thick at −20°C and immediately transferred to a micro slide box kept on dry ice and stored at −80°C. A new blade was used for each frozen sample.

### RNA isolation

RNA isolation of FFPE sections of human liver biopsies was performed as previously described [13]. RNA (5μg) was treated and the quality and yield were assessed by electrophoresis using the Agilent 2100 bioanalyzer (Agilent, Palo Alto, USA).

### RNA sequencing (RNA-Seq)

Libraries for RNA-Seq were prepared with Nugen Ovation Human FFPE RNA-Seq Multiplex System. The workflow consists of double-stranded cDNA generation using a mixture of random and poly (T) priming, fragmentation of double stranded cDNA, end repair to generate blunt ends, adaptor ligation, strand selection via nucleotide analog-targeted degradation, InDA-C-mediated adaptor cleavage and PCR amplification to produce the final library. Diffenrent adaptors were used for multiplexing samples in one lane. Sequencing was performed on Illumina HiSeq 2500 for a single read 50 run. Data quality check was done on Illumina SAV. Demultiplexing was performed with Illumina CASAVA 1.8.2. The gene expression level was calculated using RSEM software [36] and TPM (transcript per million) was used to normalized the gene expression.

### Expression pattern, function enrichment and network analysis of differentially expressed genes (DEGs)

The read was first mapped to the latest UCSC transcript set containing 25343 gene probe sets using Bowtie2 version 2.1.0. DEGs were identified using the Partek software. Only genes that were significantly modulated (false discovery rate (FDR)-adjusted; *p*-value <0.05) and more than a 2 fold change were considered differentially expressed in the AH livers compared with normal livers. The pathway and network analysis was performed using Ingenuity Pathway Analysis (IPA). IPA computes a score for each network according to the fit of the set of supplied focus genes. These scores indicate the likelihood of the focus genes to belong to a network versus those obtained by chance. A score >2 indicates a ≤ 99% confidence that a focus gene network was not generated by chance alone. The canonical pathways generated by IPA are the most significant for the uploaded data set. Fischer's exact test with FDR option was used to calculate the significance of the canonical pathway. The data presented here are accessible at the UCLA website (http://hpc.ucla.edu/hoffman2/file-transfer/gol.php).

### Quantitative real-time PCR

Real-time PCR was performed as previously described [13]. Primer sequences are listed in Supplementary Table S1.

### Immunohistochemical analysis

FFPE tissue slides were double stained for BRCA1, BRCA2, p15, Bax and Tec (Abcam Inc., Cambridge MA) with Ubiquitin (Millipore, Temecula, CA). A second set of slides were stained for BRCA1, BRCA2, p15, Bax and Tec (Enzo Life Sciences, Farmingdale, NY) with Ubiquitin (Millipore, Temecula, CA). BRCA1, BRCA2, p15, Bax and Tec were detected using donkey anti rabbit Alexa Fluor 488 (Jackson Immuno Research Laboratories Inc. West Grove, PA). Ubiquitin was detected using donkey anti mouse Alexa Fluor 594 (Jackson Labs. West Grove, PA). All slides were stained with the nuclear stain DAPI (Molecular Probes, Eugene, OR). The fluorescence intensity of stained protein of interest was measured quantitatively using a 40× objective and a standard exposure time of 800ms using a Nikon 400 fluorescent microscope with three filters (FITC-green, Texas Red, and Tri-Color), and the Nikon morphometric system. The results were displayed as a graph attached to the immunofluorescent photography using a screen snip.

### Protein extraction and immunoblotting

The total protein was extracted with Extraction Buffer EXB using Qproteome FFPE Tissue Kit following the instructions (Qiagen, San Diego, CA). Briefly, the excised tissues were incubated on ice for 5 min changing to 100°C for 20 min. The tissues were then incubated at 80°C for 2 h with agitation. After centrifugation, the supernatant containing the extracted proteins was transferred to a new Collection Tube and stored at −20°C. 50 μg of each extracted protein was used for PAGE. Proteins were fractionated by electrophoresis using 10% SDS-PAGE and electrophoretically transferred onto a PVDF blot membrane (Bio-Rad, Hercules, CA). Subsequent Western blot was performed as previously described [35].

### Statistical analysis

Statistical significance was determined using the t-test and One Way ANOVA test with SigmaStat software. *P* < 0.05 was considered as a statistically significant difference. Regression plots were constructed using SigmaPlot software. All data were presented as the mean ±S.E.M and were representative of at least three-independent experiments done in triplicate.

## SUPPLEMENTARY MATERIAL TABLES AND FIGURES













## Abbreviations

AH: alcoholic hepatitis
BAX: BCL2-associated X protein
BRCA1/2: breast cancer susceptibility gene 1/2
CDKN1A: cyclin-dependent kinase inhibitor 1A
CDKN2B: cyclin-dependent kinase inhibitor 2B
DDC: diethyl 1, 4-dehydro-2, 4, 6-trimethyl-3, 5-pyridine-dicarboxylate
DEG: differentially expressed genes
FFPE: formalin-fixed paraffin-embedded
IPA: ingenuity pathway analysis
MDB: Mallory-Denk body
RNA-Seq: RNA sequencing
TEC: tyrosine kinase expressed in hepatocellular carcinoma

## References

[1] Arteel GE (2003). Oxidants and antioxidants in alcohol-induced liver disease. Gastroenterology.

[2] Sancar A, Lindsey-Boltz LA, Unsal-Kacmaz K, Linn S (2004). Molecular mechanisms of mammalian DNA repair and the DNA damage checkpoints. Annu Rev Biochem.

[3] Koteish A, Yang S, Lin H, Huang J, Diehl AM (2002). Ethanol induces redox-sensitive cell-cycle inhibitors and inhibits liver regeneration after partial hepatectomy. Alcohol Clin Exp Res.

[4] French BA, Oliva J, Bardag-Gorce F, Li J, Zhong J, Buslon V, French SW (2012). Mallory-denk bodies form when ezh2/h3k27me3 fails to methylate DNA in the nuclei of human and mice liver cells. Exp Mol Pathol.

[5] Sherr CJ, Roberts JM (2004). Living with or without cyclins and cyclin-dependent kinases. Genes Dev.

[6] Park IK, Qian D, Kiel M, Becker MW, Pihalja M, Weissman IL, Morrison SJ, Clarke MF (2003). Bmi-1 is required for maintenance of adult self-renewing haematopoietic stem cells. Nature.

[7] Lelbach WK (1975). Cirrhosis in the alcoholic and its relation to the volume of alcohol abuse. Ann N Y Acad Sci.

[8] Haybaeck J, Stumptner C, Thueringer A, Kolbe T, Magin TM, Hesse M, Fickert P, Tsybrovskyy O, Muller H, Trauner M, Zatloukal K, Denk H (2012). Genetic background effects of keratin 8 and 18 in a ddc-induced hepatotoxicity and mallory-denk body formation mouse model. Lab Invest.

[9] French SW, Bardag-Gorce F, French BA, Li J, Oliva J (2011). The role of innate immunity in the pathogenesis of preneoplasia in drug-induced chronic hepatitis based on a mouse model. Exp Mol Pathol.

[10] Yuan QX, Marceau N, French BA, Fu P, French SW (1996). Mallory body induction in drug-primed mouse liver. Hepatology.

[11] Oliva J, Bardag-Gorce F, French BA, Li J, McPhaul L, Amidi F, Dedes J, Habibi A, Nguyen S, French SW (2008). Fat10 is an epigenetic marker for liver preneoplasia in a drug-primed mouse model of tumorigenesis. Exp Mol Pathol.

[12] Li J, Bardag-Gorce F, Dedes J, French BA, Amidi F, Oliva J, French SW (2008). S-adenosylmethionine prevents mallory denk body formation in drug-primed mice by inhibiting the epigenetic memory. Hepatology.

[13] Liu H, Li J, Tillman B, French BA, French SW (2014). Ufmylation and fatylation pathways are downregulated in human alcoholic and nonalcoholic steatohepatitis, and mice fed ddc, where mallory-denk bodies (mdbs) form. Exp Mol Pathol.

[14] Liu H, Li J, Tillman B, Morgan TR, French BA, French SW (2014). Tlr3/4 signaling is mediated via the nfkappab-cxcr4/7 pathway in human alcoholic hepatitis and non-alcoholic steatohepatitis which formed mallory-denk bodies. Exp Mol Pathol.

[15] Xiong Y, Chen X, Chen Z, Wang X, Shi S, Zhang J, He X (2010). Rna sequencing shows no dosage compensation of the active x-chromosome. Nat Genet.

[16] Wang Z, Gerstein M, Snyder M (2009). Rna-seq: A revolutionary tool for transcriptomics. Nat Rev Genet.

[17] Yuan QX, Marceau N, French BA, Fu P, French SW (1995). Heat shock in vivo induces mallory body formation in drug primed mouse liver. Exp Mol Pathol.

[18] Caldwell S, Ikura Y, Dias D, Isomoto K, Yabu A, Moskaluk C, Pramoonjago P, Simmons W, Scruggs H, Rosenbaum N, Wilkinson T, Toms P, Argo CK (2010). Hepatocellular ballooning in nash. J Hepatol.

[19] Oliva J, Bardag-Gorce F, Li J, French BA, Nguyen SK, Lu SC, French SW (2009). Betaine prevents mallory-denk body formation in drug-primed mice by epigenetic mechanisms. Exp Mol Pathol.

[20] Awad MM, Sanders JA, Gruppuso PA (2000). A potential role for p15(ink4b) and p57(kip2) in liver development. FEBS Lett.

[21] Subramanian A, Tamayo P, Mootha VK, Mukherjee S, Ebert BL, Gillette MA, Paulovich A, Pomeroy SL, Golub TR, Lander ES, Mesirov JP (2005). Gene set enrichment analysis: A knowledge-based approach for interpreting genome-wide expression profiles. Proc Natl Acad Sci U S A.

[22] Affo S, Dominguez M, Lozano JJ, Sancho-Bru P, Rodrigo-Torres D, Morales-Ibanez O, Moreno M, Millan C, Loaeza-del-Castillo A, Altamirano J, Garcia-Pagan JC, Arroyo V, Gines P (2013). Transcriptome analysis identifies tnf superfamily receptors as potential therapeutic targets in alcoholic hepatitis. Gut.

[23] Yi YW, Kang HJ, Bae I (2014). Brca1 and oxidative stress. Cancers (Basel).

[24] Ouchi T, Lee SW, Ouchi M, Aaronson SA, Horvath CM (2000). Collaboration of signal transducer and activator of transcription 1 (stat1) and brca1 in differential regulation of ifn-gamma target genes. Proc Natl Acad Sci U S A.

[25] Oliva J, Bardag-Gorce F, Lin A, French BA, French SW (2010). The role of cytokines in ubd promoter regulation and mallory-denk body-like aggresomes. Exp Mol Pathol.

[26] Diehl JA, Ponugoti B (2010). Ubiquitin-dependent proteolysis in g1/s phase control and its relationship with tumor susceptibility. Genes Cancer.

[27] Harbour JW, Luo RX, Dei Santi A, Postigo AA, Dean DC (1999). Cdk phosphorylation triggers sequential intramolecular interactions that progressively block rb functions as cells move through g1. Cell.

[28] Aravinthan A, Pietrosi G, Hoare M, Jupp J, Marshall A, Verrill C, Davies S, Bateman A, Sheron N, Allison M, Alexander GJ (2013). Hepatocyte expression of the senescence marker p21 is linked to fibrosis and an adverse liver-related outcome in alcohol-related liver disease. PLoS One.

[29] Havens CG, Walter JC (2011). Mechanism of crl4(cdt2), a pcna-dependent e3 ubiquitin ligase. Genes Dev.

[30] Fataccioli V, Andraud E, Gentil M, French SW, Rouach H (1999). Effects of chronic ethanol administration on rat liver proteasome activities: Relationship with oxidative stress. Hepatology.

[31] French SW, Bardag-Gorce F, Li J, French BA, Oliva J (2010). Mallory-denk body pathogenesis revisited. World J Hepatol.

[32] Shin S, Wolgamott L, Yu Y, Blenis J, Yoon SO (2011). Glycogen synthase kinase (gsk)-3 promotes p70 ribosomal protein s6 kinase (p70s6k) activity and cell proliferation. Proc Natl Acad Sci U S A.

[33] Zwolanek F, Riedelberger M, Stolz V, Jenull S, Istel F, Koprulu AD, Ellmeier W, Kuchler K (2014). The non-receptor tyrosine kinase tec controls assembly and activity of the noncanonical caspase-8 inflammasome. PLoS Pathog.

[34] Peng Y, French BA, Tillman B, Morgan TR, French SW (2014). The inflammasome in alcoholic hepatitis: Its relationship with mallory-denk body formation. Exp Mol Pathol.

[35] Liu H, Gong M, French BA, Li J, Tillman B, French SW (2014). Mallory-denk body (mdb) formation modulates ufmylation expression epigenetically in alcoholic hepatitis (ah) and non-alcoholic steatohepatitis (nash). Exp Mol Pathol.

[36] Li B, Dewey CN : Rsem (2011). Accurate transcript quantification from rna-seq data with or without a reference genome. BMC Bioinformatics.

