# Supplementary material for: Aberrant modulation of the BRCA1 and G1/S cell cycle pathways in alcoholic hepatitis patients with Mallory Denk Bodies revealed by RNA sequencing
Source: Oncotarget. 2015 Nov 25;6(40):42491–503. doi: 10.18632/oncotarget.6382 (PMC4767447; doi:10.18632/oncotarget.6382)
Supplement: Supplementary file 1 [file oncotarget-06-42491-s001.pdf]

Aberrant modulation of the BRCA1 and G1/S cell cycle pathways in alcoholic hepatitis patients with Mallory Denk Bodies revealed by RNA sequencing

Supplementary material

Table S1. Sets of specific oligonucleotide quantitative real-time PCR (qRT-PCR) primers

Table S2. Upregulated genes in AH livers with MDBs

Table S3. Downregulated genes in AH livers with MDBs

Table S4. The canonical pathways in AH livers with MDBs

Table S5. Gene network in AH livers with MDBs

Table S6. Classification of genes disease and function in AH livers with MDBs

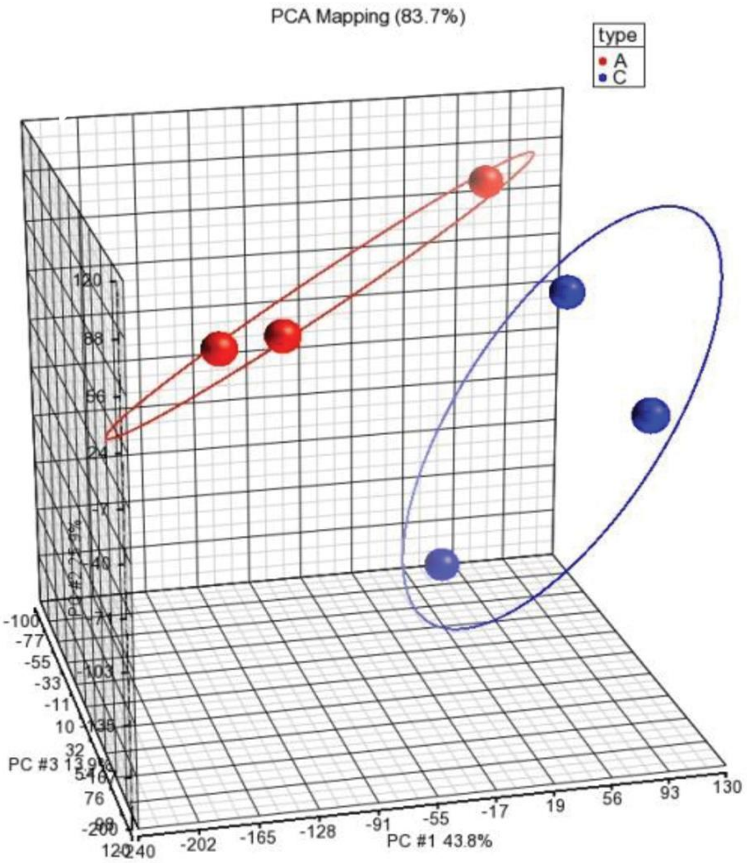

Supplementary Fig. 1: The Principal Component Analysis (PCA) of the samples. The PCA Mapping shows the three samples in the AH group cluster together and the three samples in the control group cluster together. Red represents AH group and blue represents control group. Each dot represents each sample.

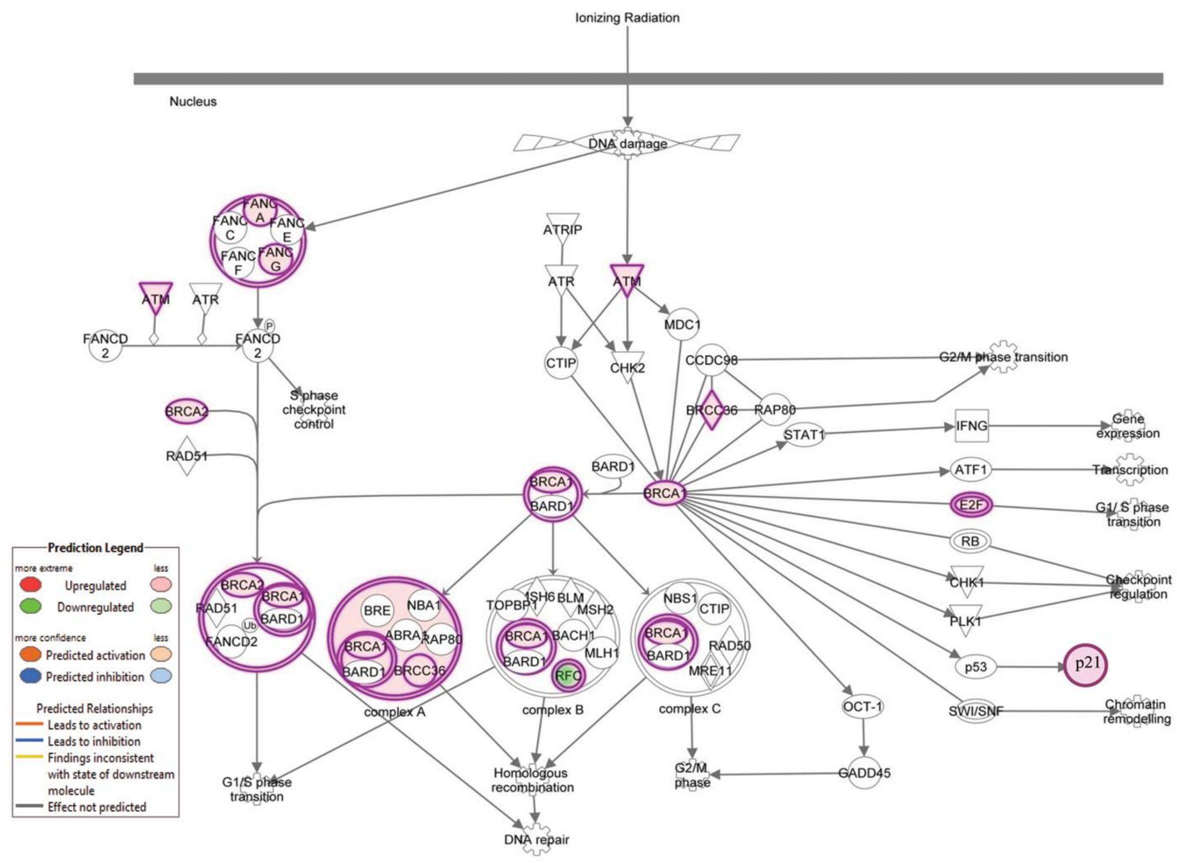

Supplementary Fig. 2: Schematic diagram of the activated BRCA1-mediated signaling pathway derived using IPA. Molecules are represented as nodes and the biological relationship between two nodes is represented as an edge (line). Nodes are displayed using various shapes that represent the functional class of gene product. The genes with relative higher expression in AH livers vs normal livers are depicted in red.

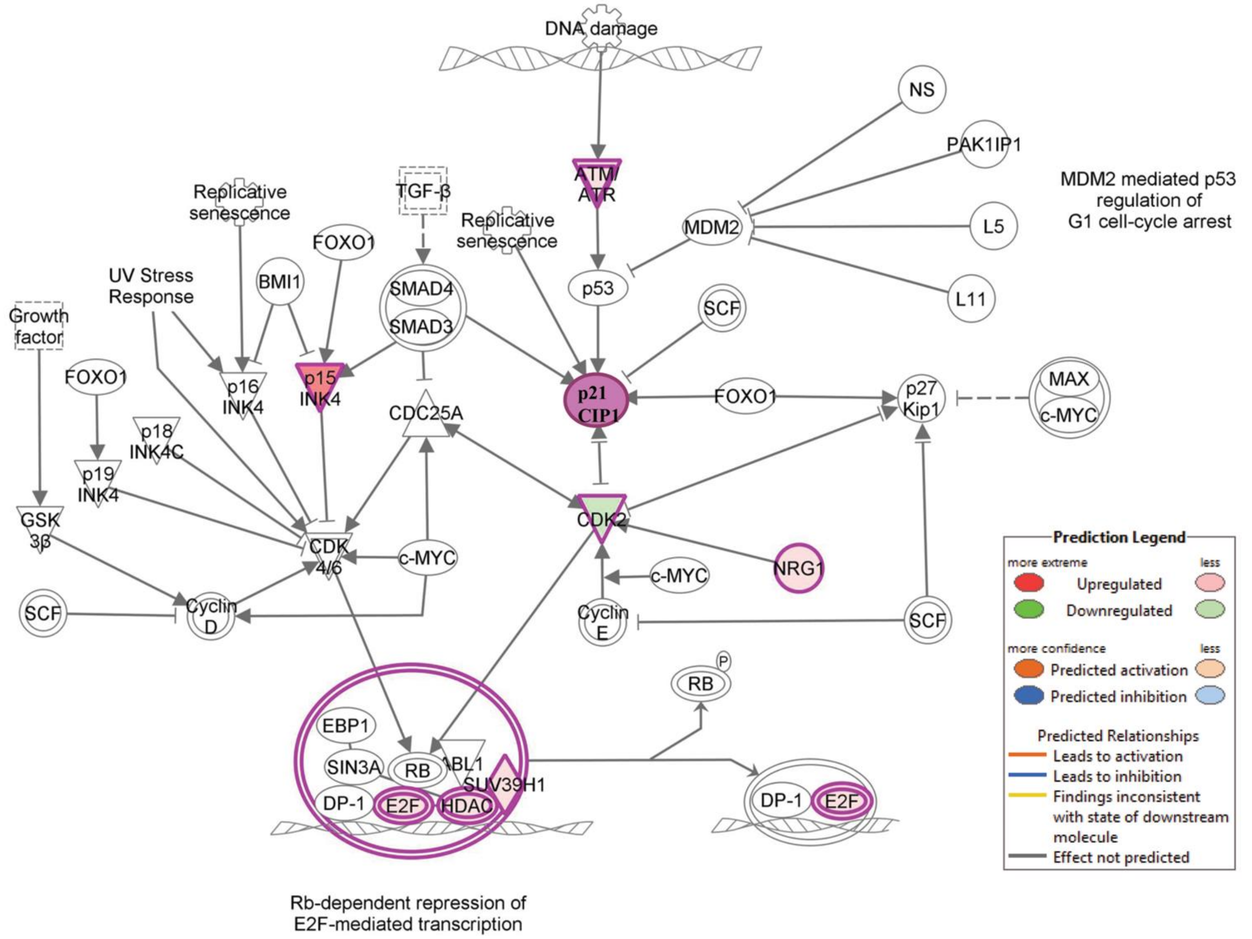

Supplementary Fig. 3: Hyperactivation of G1/S cell cycle checkpoint in AH livers with MDBs. The RNA-Seq analysis illustrate DEGs with red (up regulation) and green (down regulation) between the AH livers and normal livers. All other genes had no obvious differences in this experiment. Altered modulation of G1/S checkpoint regulation was shown at the transcriptional level in AH livers compared with normal livers.

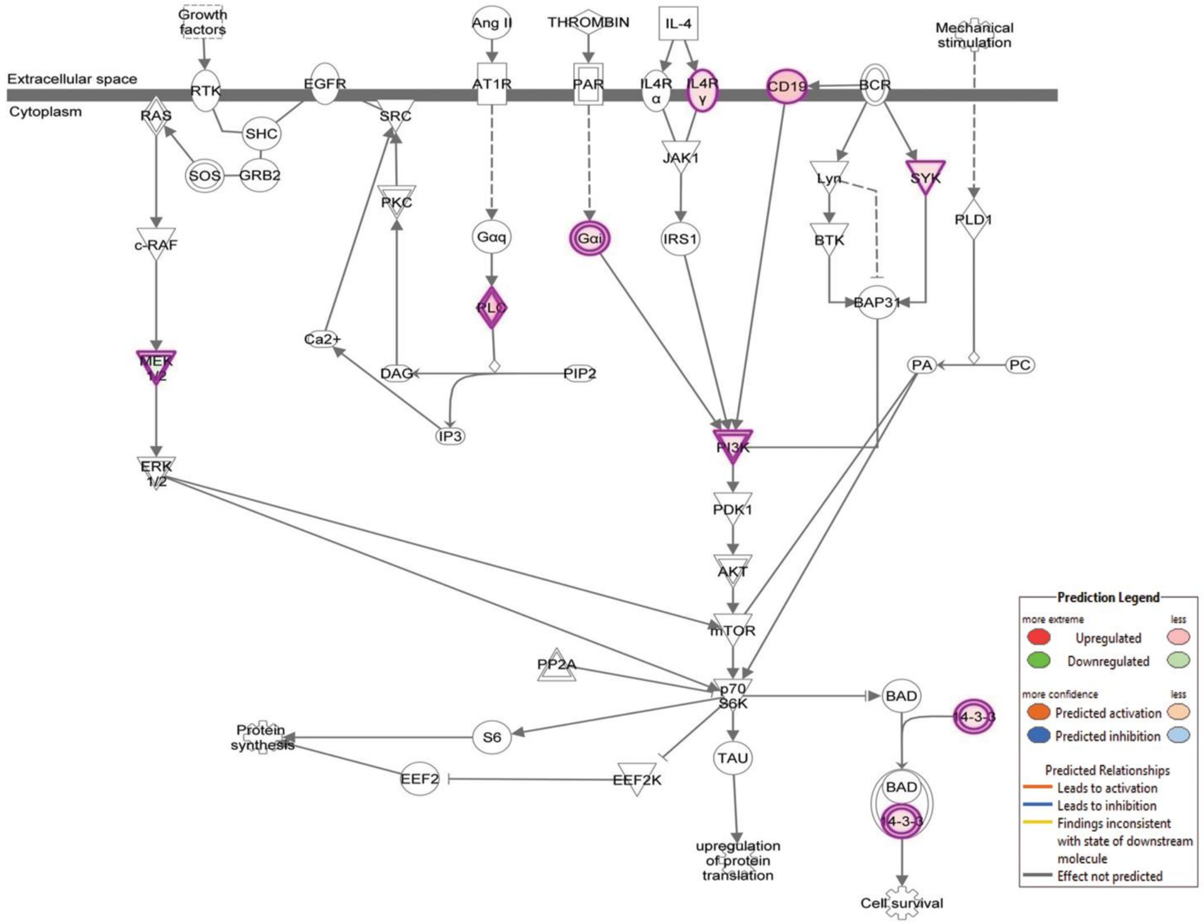

Supplementary Fig. 4: Schematic diagram of activated p70S6K signaling pathway derived using IPA.

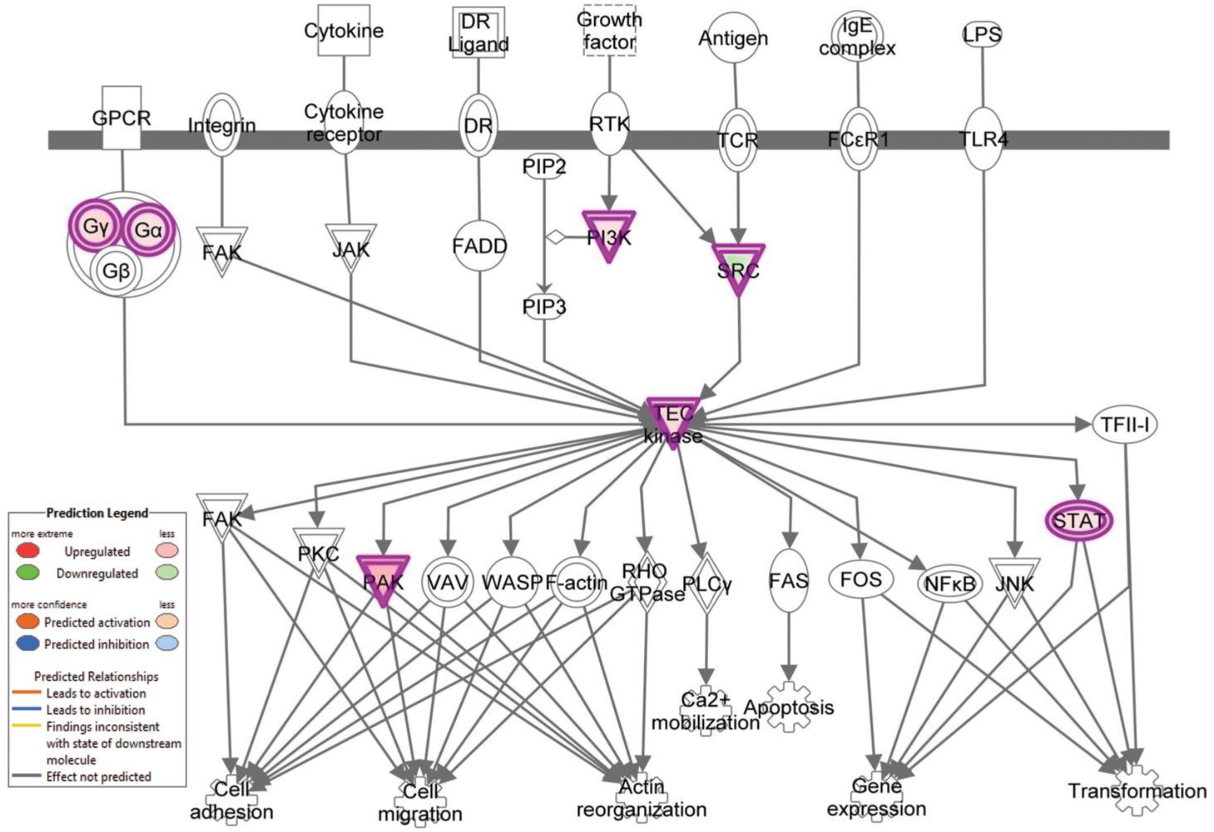

Supplementary Fig. 5: Schematic diagram of activated Tec kinase signaling pathway derived using IPA.

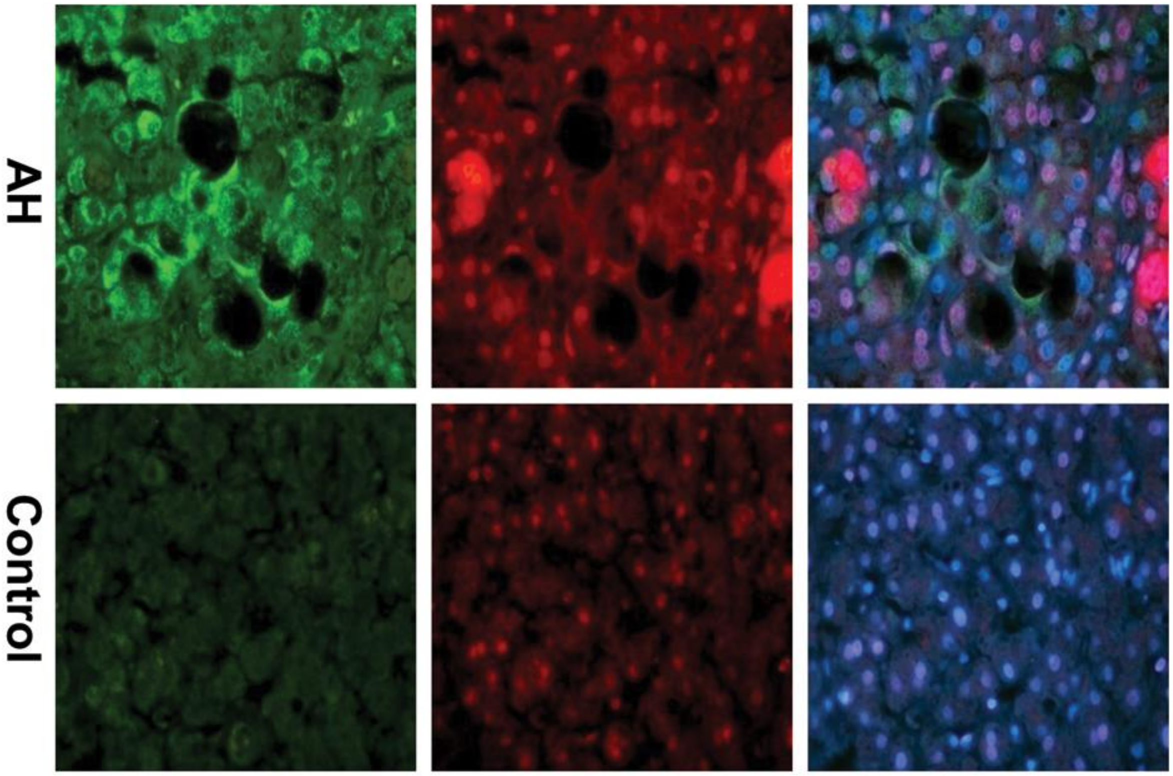

Supplementary Fig. 6: Immunohistochemical stains for Tec. The liver sections from AH livers and controls were double stained with antibodies to Tec (green), Ub (red) and DAP1 (blue). The MDBs stained positive for both Ub and Tec. ×520

Supplementary Table S1

Sets of specific oligonucleotide quantitative real-time PCR (qRT-PCR) primers.

Gene name (Species)	Accession Number	Sequences of primers	
BRCA1	NM_007294	Forward Primer	5'-AACCCCTTACCTGGAATCTG-3'
(Human)		Reverse Primer	5'-TCCCTGCTCACACTTTCTTC-3'
BRCA2	NM_000059	Forward Primer	5'-TGCTGTTAAGGCCCAGTTAG-3'
(Human)		Reverse Primer	5'-GCAGAGGAAAAGGTCTAGGG-3'
BRCC3	NM_024332	Forward Primer	5'-TGTGCCTTGAATCAGCAGTA-3'
(Human)		Reverse Primer	5'-TTTTGCTCCAGTCTGTCCTC-3'
CDKN2B (p15)	NM_004936	Forward Primer	5'-CGGAAATCCCCTTATGACTT-3'
(Human)		Reverse Primer	5'-CACTGAGTTTGCAACAGTGC-3'
TEC	NM_003215	Forward Primer	5'-TGTTAGCTGATGCCAGTTGA-3'
(Human)		Reverse Primer	5'-TATCCAAGATGGGTTCCTGA-3'
GNG2	NM_053064	Forward Primer	5'-GGCTCTTTTGTCCACTGAGA-3'
(Human)		Reverse Primer	5'-ACCATATCTTGGGGAAGGAG-3'
PLCD3	NM_133373	Forward Primer	5'-CCTCCCACAACACCTATCTG-3'
(Human)		Reverse Primer	5'-TCTCCAGGGATAGGATGACA-3'
CDKN1A (p21)	NM_000389	Forward Primer	5'-AAGACCATGTGGACCTGTCA-3'
(Human)		Reverse Primer	5'-TAGGGCTTCCTCTTGGAGAA-3'
CXCL14	NM_004887	Forward Primer	5'-GGAGCAGGTCTCTCTGCATC-3'
(Human)		Reverse Primer	5'-GTTGGGAACCTCACATGCTT-3'
BAX	NM_138761	Forward Primer	5'-GCTGGACATTGGACTTCCTC-3'
(Human)		Reverse Primer	5'-CTCAGCCCATCTTCTTCCAG-3'
BCL-2	NM_000633	Forward Primer	5'-AGATGGGAACACTGGTGGAG-3'
(Human)		Reverse Primer	5'-CTTCCCCAAAAGAAATGCAA-3'
PI3K	NM_006219	Forward Primer	5'-CATGGATTCCAAAATGAAGC-3'
(Human)		Reverse Primer	5'-CAACATCCGAAGATCCAAAC-3'
ATM	NM_000051	Forward Primer	5'-ACTGCCAAGGACAAATGAGG-3'
(Human)		Reverse Primer	5'-TGAGCAACTGACTGGCAAAC-3'
MAP2K1	NM_002755	Forward Primer	5'-AAGCAGAAGGTGGGAGAACT-3'
(Human)		Reverse Primer	5'-AGAACCTGCAGCTCCCTTAT-3'
STAT5A	NM_003152	Forward Primer	5'- GAACCCTGACCATGTACTCG-3'
(Human)		Reverse Primer	5'- TCTGGCAGAGGTGAAAAGAC-3'
E2F3	NM_001949	Forward Primer	5'-GATGATGTAACAGCCCCAAG-3'
(Human)		Reverse Primer	5'-GCTTAAGGAAATGCCACTCA-3'
NOX4	NM_016931	Forward Primer	5'-TCAAGACTCCGAAATTCTGC-3'
(Human)		Reverse Primer	5'-GTTTCCAGTCATCCAACAGG-3'
UBE2E2	NM_152653	Forward Primer	5'- GAAGTCTTTATTGGTGGGTG-3'
(Human)		Reverse Primer	5'- CGGGAGGGACAGAAGGCTAG-3'
BRCA1	NM_009764	Forward Primer	5'-AGTGCCAGTGTCAAGGAGAG-3'
(Mouse)		Reverse Primer	5'-ACTCCTTTCCTGGTGGAATC-3'
BRCA2	NM_001081001	Forward Primer	5'-ACGAGATTGATGACCCAAAA-3'
(Mouse)		Reverse Primer	5'-TCTTCTGAAATGGCGTTCTC-3'
CDKN2B (p15)	NM_007670	Forward Primer	5'- CATTTTCTGCAGCTGGATCT-3'
(Mouse)		Reverse Primer	5'- TTCACAGGGGAAGGTACTGA-3'
CDKN1A (p21)	NM_007669	Forward Primer	5' - TGCTCAGACCTGTGAAGACA-3'
(Mouse)		Reverse Primer	5'- CTTCCAGTCCACTGAGCTGT-3'
TEC	NM_001113460	Forward Primer	5'-ATGCTGCTAAGCATGTGTCA-3'
(Mouse)		Reverse Primer	5'-TTAAACACTTCTGGGGGACA-3'
GNG2	NM_010315	Forward Primer	5'- AGTCTCTGAAGACCCCATCC-3'
(Mouse)		Reverse Primer	5'- GAACTTCTTCTCCCGAAAGG-3'
BAX	NM_007527	Forward Primer	5'- TAGCAAACTGGTGCTCAAGG-3'
(Mouse)		Reverse Primer	5'- ATGGTCACTGTCTGCCATGT-3'
Ki67	NM_001081117	Forward Primer	5'-CTCCACGAACCTCAAAGAGA-3'
(Mouse)		Reverse Primer	5'-TGTGGATTCCTTCACACCTT-3'
ATM	NM_007499	Forward Primer	5'- TGGTTGTGACAGTCTGATGG-3'
(Mouse)		Reverse Primer	5'- GCAGTTACAGATCGGCCTAA-3'
CDK2	NM_183417	Forward Primer	5'- TGGGCTGCAAGTACTACTCC-3'

(Mouse)	Reverse Primer	5'- TTGTGATGCAGCCACTTCTA-3'
